# Discovery of Oxazol-2-amine Derivatives as Potent Novel FLT3 Inhibitors

**DOI:** 10.3390/molecules25215154

**Published:** 2020-11-05

**Authors:** Hyo Jeong Kim, Hwani Ryu, Jie-Young Song, Sang-Gu Hwang, Shivakumar S. Jalde, Hyun-Kyung Choi, Jiyeon Ahn

**Affiliations:** 1 Division of Radiation Biomedical Research, Korea Institute of Radiological & Medical Sciences, Seoul 01812, Korea; hjkim@kirams.re.kr (H.J.K.); hwanya85@kirams.re.kr (H.R.); immu@kirams.re.kr (J.-Y.S.); sgh63@kirams.re.kr (S.-G.H.); 2 Department of Biology, Korea University, Seoul 02841, Korea; 3 Department of Medicinal Chemistry, Jungwon University, Goesan 28024, Korea; jaldeshivkumar641@gmail.com

**Keywords:** FLT3 inhibitor, acute myeloid leukemia, DNA damage repair, combination therapy, PARP1 inhibitor

## Abstract

Internal tandem duplication (ITD) of FMS-like tyrosine kinase 3 (FLT3) is the most common mutation in patients with acute myeloid leukemia (AML). FLT3-ITD^+^ induces constitutive activation of FLT3, causing an abnormally rapid proliferation of cancer cells. In this study, we identified novel FLT3 inhibitors and investigated 5-(4-fluorophenyl)-*N*-phenyloxazol-2-amine (compound **7**; **7c**) as candidates for the treatment of AML. The results showed that **7c** inhibited the activities of FLT3 and mutated FLT3 in a cell-free kinase assay and Molm-13 and MV4-11 cells, as well as the proliferation of FLT3-ITD^+^ AML cells, increasing apoptosis. The anti-leukemic activity of **7c** was confirmed by in vivo tumor growth inhibition in MV4-11 xenograft mice. Besides, **7c** suppressed the expression of DNA damage repair genes. Combination treatment with **7c** and olaparib (a poly (ADP-ribose) polymerase [PARP] inhibitor) synergistically inhibited cell proliferation in Molm-13 and MV4-11 cells. Our findings demonstrated that **7c** is a therapeutic candidate targeting FLT3 for AML treatment and suggested that combination treatment with **7c** and a PARP inhibitor may be an effective therapy regimen for FLT3-mutated AML.

## 1. Introduction

Acute myeloid leukemia (AML) is a heterogeneous malignancy characterized by rapid clonal proliferation or poor differentiation of myeloid lineage cells in the hematopoietic system. The incidence of AML increases with age and occurs mainly in adults, accounting for nearly 80% of AML cases [1]. The standard therapeutic protocol for AML is combination treatment with cytarabine for 7–10 days and daunorubicin for 3 days (“7 + 3” regimen) with or without hematopoietic stem cell transplantation (HSCT) [2,3,4]. However, the “7 + 3” regimen is mostly beneficial for patients younger than 60 years who show a 5-year overall survival (OS) of 40%, but not for patients older than 60 years (OS, 5–15%), mainly due to underlying health conditions [5].

Genomic studies on AML have identified several recurrently mutated genes that enable its classification and diagnosis using predictive biomarkers to help develop anti-leukemic agents. One of the most frequently mutated genes in AML is the FMS-like tyrosine kinase 3 receptor gene (*FLT3*), a well-characterized oncogenic mutation; it occurs in approximately 30% of patients. Approximately 25% of patients with AML have an internal tandem duplication (ITD), whereas 5–10% of patients with AML have point mutations in the tyrosine kinase domain (TKD), typically at codon D835 [6,7]. FLT3 is a member of class III receptor tyrosine kinase family that is activated by binding to FLT3 ligands, and it induces the proliferation and differentiation of hematopoietic precursor cells. However, FLT3-ITD and FLT3-TKD can constitutively activate FLT3 and induce rapid cell proliferation via the activation of its downstream signaling molecules, including the signal transducer and activator of transcription 5 (STAT5).

After decades of standard treatments, the U.S. Food and Drug Administration (FDA) approved FLT3-targeting therapeutic agents, allowing targeted therapy for FLT3-mutated AML. First-generation FLT3 inhibitors, such as sunitinib, midostaurin, lestaurtinib, sorafenib, ponatinib, and tandutinib, are multi-kinase inhibitors approved by the FDA in 2017 for the treatment of FLT3-mutated AML. Inhibition of multiple RTKs induces AML cell death via the inhibition of FLT3; however, these inhibitors are cytotoxic to healthy cells owing to unclear target specificity. Second-generation FLT3 inhibitors, such as KW-2449, crenolanib, gilteritinib, and quizartinib, which mainly target FLT3 mutants, have relatively low toxicity [8]. Of these, quizartinib is a more potent and selective FLT3 inhibitor than midostaruin, but the FDA declined it in 2019. Thus, there is a need to develop novel inhibitors targeting FLT3.

Previous studies suggest that FLT3-ITD^+^ AML cells generate high levels of reactive oxygen species (ROS) that induce DNA damage, mutations, double-strand DNA breaks (DSB), and chromosomal instability, but they can survive owing to enhanced DNA repair activities [9,10,11,12]. Interestingly, FLT3 inhibitors downregulate DNA repair genes of the homologous recombination (HR) and nonhomologous end-joining (NHEJ) repair pathways. PARP1 is an abundant nuclear protein that acts as an early DNA damage sensor to rapidly recruit DNA repair enzymes to single-strand DNA break or DSB sites by adding ADP-ribose polymers to lysine residues and target proteins, a process known as poly(ADP-ribosyl)ation (PARylation) [13,14]. Therefore, an FLT3 inhibitor-mediated “BRCAness/DNA-PKness” phenotype can induce synthetic lethality with a PARP inhibitor in combination therapy.

In this study, to identify tyrosine kinase inhibitors targeting FLT3, we designed and synthesized 11 oxazol-2-amine-based small-molecule compounds. We aimed to investigate the anti-leukemic effect of the novel compounds and the combination therapeutic effect of the novel FLT3 compound with a PARP1 inhibitor in FLT3-ITD^+^ AML cells. Our study will provide additional data for the use of FLT3 inhibitors in AML treatment protocols.

## 2. Results

### 2.1. **7c** Has Anti-Leukemic Activity in AML Cells Both In Vitro and In Vivo

**Scheme 1:**
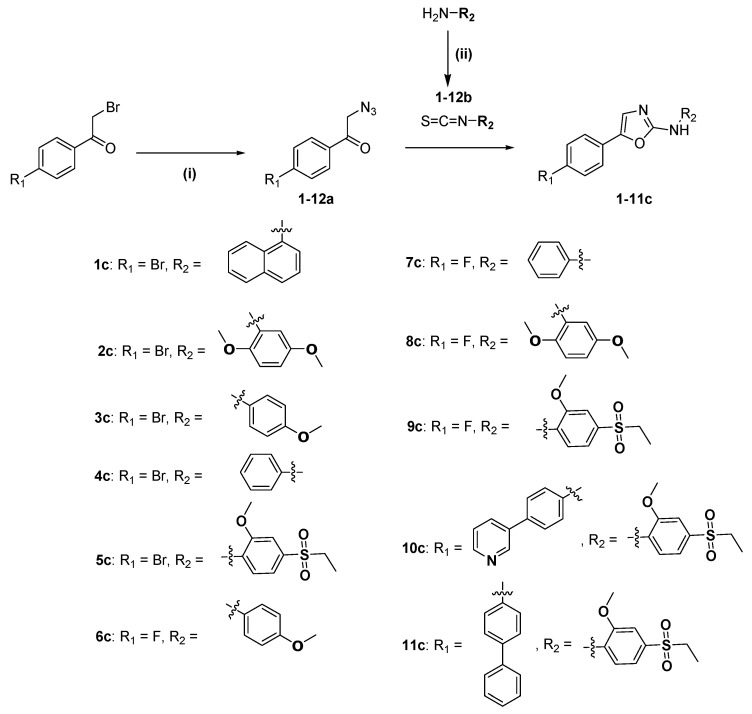
Reagents and conditions: (**i**) sodium azide, acetone, H_2_O room temperature for 16 h; (**ii**) thiophosgene, dichloromethane for 4 h; and (**iii**) PPh_3_, dioxane 90 °C for 4 h.

**Figure 1:**
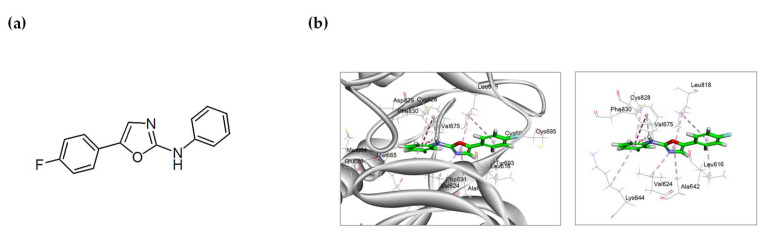
Structure of synthesized compound **7** (**7c**). (**a**) Chemical structure of **7c**. (**b**) 3D binding mode and interaction of **7c** with FMS-like tyrosine kinase 3 (FLT3). Green, **7c**; pink dotted line, pi–alkyl bond. Relevant distances are expressed in Å.

Of the eleven synthesized compounds (Scheme 1 and Table 1), **10c**, **11c**, **5c**, **2c**, **1c**, **6c**, and **4c** showed the highest FLT3 inhibitory activity at 100 nM. Of the eight compounds that showed the inhibitory kinase activity against FLT3-ITD and FLT3-D835Y in vitro, five compounds (**7c, 5c, 4c, 2c,** and **6c**) inhibited the growth of Molm-13 and MV4-11 cells at 100 nM, but not that of AML HL-60 (FLT3-null) cells at 10,000 nM (Table 1) and human chronic myeloid leukemia K562 cells (FLT3 WT) at 1000 nM (Figure S1). Overall, **7c** (5-(4-fluorophenyl)-*N*-phenyloxazol-2-amine) was the most effective in inhibiting the growth of Molm-13 and MV4-11 cells and was further examined as a novel putative FLT3 inhibitor (Figure 1a, Table 2, and Figure S2).

**Figure 2:**
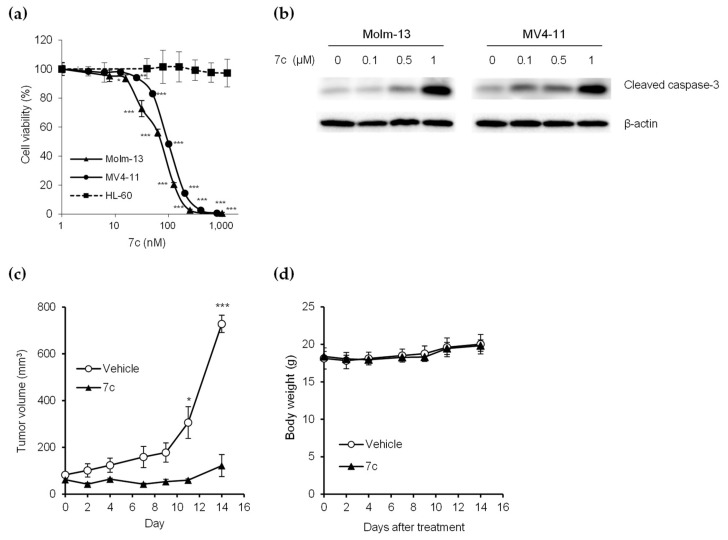
Compound **7c** inhibited FMS-like tyrosine kinase 3-internal tandem duplication-positive acute myeloid leukemia (FLT3-ITD^+^ AML) cells. (**a**) Human FLT3-ITD^+^ AML cells (Molm-13 and MV4-11) and FLT3-null AML cells (HL-60) were treated with **7c** at the indicated concentrations for 5 days and viable cells were evaluated using the CCK-8 assay. (**b**) Cell lysates were prepared at 48 h after treatment with **7c** and determined by immunoblotting for cleaved caspase-3. β-actin was used as the loading control. (**c**) MV4-11 cells mixed with Matrigel were subcutaneously injected into the right hind leg thigh of BALB/c nu/nu mice (*n* = 5/vehicle control group, *n* = 5; treatment group, *n* = 10). Tumor size was measured on the indicated day after the first **7c** intraperitoneal administration. Tumor volume (mm^3^) was calculated as follows: (longest axis × shortest axis of tumor)^2^/2. (**d**) Body weight of MV4-11 tumor xenograft mice was determined twice weekly during the experiments. Significant difference was defined as * at *p* < 0.05, ** at *p* < 0.01, and *** at *p* < 0.001.

To understand the interaction of **7c** and FLT3, an in silico docking study was performed based on the crystal structure of FLT3 (PDB code: 4XUF) [15]. The aromatic ring of **7c** showed a pi–alkyl interaction with Val^675^, Lys^644^, Cys^828^, Leu^818^, Val^624^, Ala^642^, and Leu^616^, as well as a pi–pi T-shaped interaction with Phe^830^ (Figure 1b). The results showed that **7c** could inhibit the constitutive activation of FLT3 through direct binding, affecting AML cell growth. Consequently, **7c** at 100 nM concentration significantly inhibited 65.2% and 51.7% of Molm-13 and MV4-11 proliferation, respectively (Figure 2a and Table 1). We further investigated whether **7c** treatment induced the apoptosis of Molm-13 and MV4-11 cells and observed that it increased the cleavage of caspase-3 in both cell lines (Figure 2b). In vivo, **7c** treatment significantly inhibited MV4-11-derived tumor growth by 83.3% compared with the control (Figure 2c). Besides, we measured the body weight of mice to determine **7c** toxicity and did not observe any significant differences between the **7c**-treated and control groups (Figure 2d). Overall, **7c** exhibited anti-leukemic activity both in vitro and in vivo.

### 2.2. **7c** Inhibited the FLT3 Kinase Activity in AML Cells

**Figure 3:**
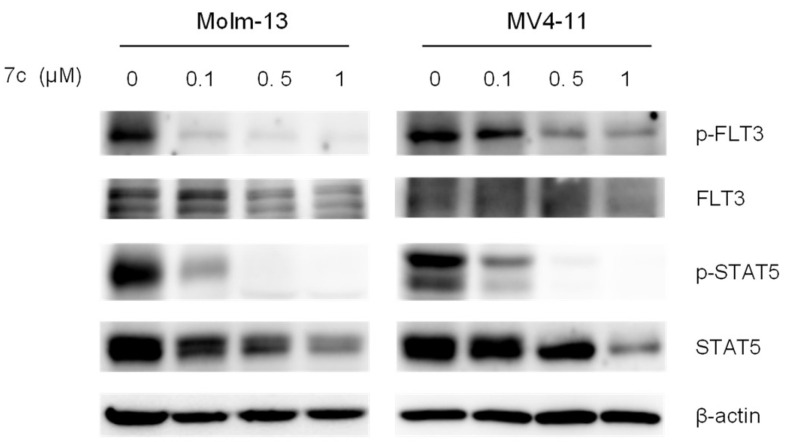
Compound **7c** inhibited FMS-like tyrosine kinase 3 (FLT3) signaling. Human FLT3-internal tandem duplication-positive acute myeloid leukemia (FLT3-ITD^+^ AML) cells (Molm-13 and MV4-11) were treated with **7c** at the indicated concentrations for 48 h, and cell lysates were subjected to immunoblotting for detecting FLT3 and STAT5. β-actin was used as the loading control.

FLT3-ITD activates FLT3 in the absence of FLT3 ligands and causes aberrant activation of STAT5 with phosphorylation in downstream signaling, leading to rapid cell proliferation and malignancy. We investigated whether **7c** inhibited FLT3 signaling in FLT3-ITD^+^ Molm-13 and MV4-11 cells in vitro. Consistent with the results of the in vitro kinase assay, phosphorylated FLT3 was inhibited by **7c** treatment in a concentration-dependent manner in both cell lines. Besides, we also observed that **7c** inhibited FLT3-downstream effector STAT5 phosphorylation and total STAT5 protein in Molm-13 and MV4-11 cells (Figure 3). However, **7c** did not regulate the phosphorylation of STAT5 in HL-60 cells (Figure S3). Thus, **7c** could be used as an FLT3 inhibitor using a cell-free kinase assay and a cell-based assay.

### 2.3. **7c** Suppressed DNA Damage Repair

**Figure 4:**
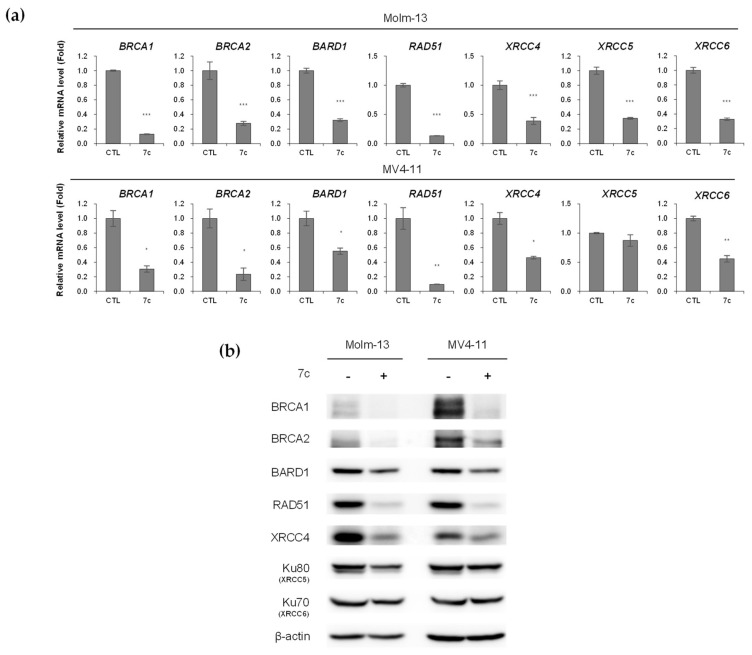
Compound **7c** inhibited DNA damage repair. (**a**) Human FMS-like tyrosine kinase 3-internal tandem duplication-positive acute myeloid leukemia (FLT3-ITD^+^ AML) cells (Molm-13 and MV4-11) were treated with 1 μM **7c** for 18 h to quantify the mRNA expression of the indicated genes by qRT-PCR. Data are from one representative experiment that repeated in triplicate. (**b**) Cell lysates were prepared 24 h after treatment with **7c** and subjected to immunoblotting for detecting the indicated proteins. Data are from one representative experiment that has three replicates. Significant difference was defined as * at *p* < 0.05, ** at *p* < 0.01, and *** at *p* < 0.001.

Previous studies suggested that FLT3-ITD increases the ROS levels in AML cells, inducing DNA damage, mutations, and chromosomal instability [9,10]. Inhibition of FLT3-ITD function by quizartinib reduces two major DSB repair activities with homologous recombination (HR) and non-homologous end-joining (NHEJ). Our previous study supported that quizartinib and the novel FLT3 inhibitor AIU2001 downregulate DNA repair HR and NHEJ genes [10,11]. To investigate whether **7c**-induced AML cell death was caused by the inhibition of DNA damage repair pathways, we determined the expression levels of HR and NHEJ genes in Molm-13 and MV4-11 cells. The results showed that **7c** significantly downregulated the mRNA and protein levels of HR (*BRCA1*, *BRCA2*, *BARD1*, and *RAD51*) and NHEJ (*XRCC4*, *XRCC5*, and *XRCC6*) genes in both cell lines (Figure 4a,b). Since DNA repair genes and proteins are downregulated, DNA damage was determined by detection of phosphorylation on Ser139 of H2AX (γ-H2AX), which is an indicator of presence of DNA double strand damage, in Molm-13 and MV4-11 cells. We observed that **7c** induced γ-H2AX in both cells (Figure S3). These results indicated that **7c** downregulated the DNA damage repair genes and induced DNA damage, probably contributing to anti-leukemic activities and leading to AML cell death.

### 2.4. Combination Therapy of **7c** with a PARP Inhibitor Showed Synthetic Lethality

**Figure 5:**
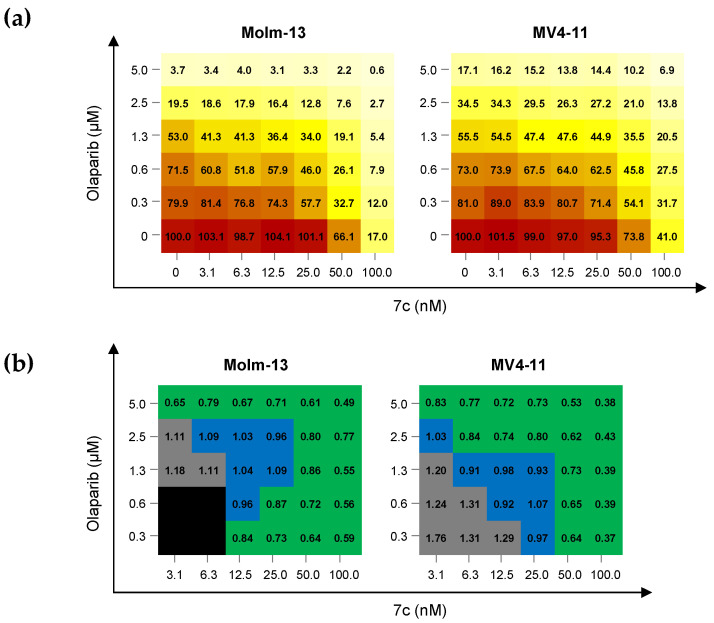
Combination treatment with compound **7c** and olaparib synergistically inhibited cell growth. (**a**) Human FMS-like tyrosine kinase 3-internal tandem duplication-positive acute myeloid leukemia (FLT3-ITD^+^ AML) cells (Molm-13 and MV4-11) were treated with **7c** or olaparib or both at the indicated concentrations. Cell viability was determined using the CCK-8 assay 5 days post treatment. Relative viability is shown for each combination at the indicated concentrations. Data are from one representative experiment that had four replicates. (**b**) Combination index (CI) scores of **7c** and olaparib combination at the indicated concentrations in Molm-13 and MV4-11 cells. Green, synergistic (CI < 0.9); blue, additive (1.1≥ CI ≥ 0.9); gray, antagonistic (CI > 1.1); black, non-significant. Each CI score represents average data derived from the indicated concentrations of single and combined compound treatments in four independent experiments.

We investigated whether **7c** could sensitize FLT3-ITD^+^ AML cells to olaparib (PARP inhibitor) in combination therapy. Olaparib is the first PARP inhibitor targeting mainly PARP 1 and 2 approved by the FDA to treat advanced ovarian cancer patients with germline BRCA mutations and is widely studied in combination with DNA damage-induced drugs or radiotherapy. Our results showed that **7c** and olaparib exerted synergistic growth inhibition in Molm-13 and MV4-11 cells (Figure 5) and that **7c** could sensitize FLT3-ITD^+^AML cells to PARP inhibition. Therefore, combination treatment with **7c** and a PARP inhibitor might increase the effectiveness of AML therapy. We observed synergistic growth inhibition at a wide range of concentrations of **7c** and olaparib in both cell lines (Figure 5b), indicating that **7c** could sensitize FLT3-ITD^+^ AML cells to PARP inhibition. Therefore, combination treatment with **7c** and a PARP inhibitor would be more effective for anti-leukemic therapy.

## 3. Discussion

In the present study, we identified novel kinase inhibitors targeting FLT3 using the in vitro kinase and cell viability assays in AML cell lines. Of the 11 synthesized compounds, **7c** was found to be a potent FLT3 inhibitor with anti-leukemic effects in vitro and in vivo. Although **5c, 10c**, and **11c** showed excellent inhibitory efficacy at 100 nM concentration against FLT3, FLT3-D835Y, and FLT3-ITD in vitro, significant growth inhibition was not observed in FLT-ITD-positive Molm-13 and MV4-11 cell lines. To identify contradictory efficacy results between cell-free and cell-based assays, we conducted an in silico physicochemical character analysis. The results showed that the pKa value of **7c** is similar to that of quizartinib, which was used as the reference, whereas that of **5c**, **10c**, and **11c** was relatively low (Table S1), probably owing to differences in chemical characteristics such as intracellular drug dissolution, absorption, and stability [16]. Overall, **7c** is a promising therapeutic agent; however, further research is required to more thoroughly investigate its structure, activity relationship, metabolism, pharmacokinetics, and off-target profiling.

Previous studies revealed a relationship between FLT3-ITD signaling and DNA damage. The phosphorylation of STAT5 increases ROS generation through direct association with the GTP-binding protein RAC1 in FLT3-ITD^+^ AML cells [9]. Increased ROS levels trigger DNA oxidation, genomic instability, DNA DSB, and error-prone DNA damage repair, whereas the enhanced DNA repair activities allow mutagenesis and malignancy, leading to the acquisition of genomic changes and aggressive AML [17,18]. Genomic instability is a pathologic feature of FLT3-ITD^+^ AML [19]; therefore, inhibition of DNA damage repair can induce the death of FLT3-ITD^+^ AML cells. Here, we showed that **7c** inhibited FLT3-STAT5 signaling as well as HR and NHEJ DNA repair genes, probably contributing to the growth inhibition and death of FLT3-ITD^+^ AML cells. These results are consistent with those of our previous study, in which we demonstrated that the inhibition of STAT5 downregulates DNA repair [11]. Previous studies suggested that AML with genomic instability is sensitive to PARP inhibition [10,20]. Our results showed that the combination of **7c** and olaparib synergistically inhibited FLT3-ITD^+^ AML cells, revealing that an FLT3 inhibitor and a PARP inhibitor could be a promising therapeutic approach for FLT3-ITD^+^ AML.

All novel putative drugs must be validated for efficacy and off-target toxicity. The multiple class III RTK inhibitor midostaurin was approved by the FDA in 2017 for the treatment of FLT3-mutant AML, whereas the FDA rejected the selective FLT3 inhibitor quizartinib in 2019. Thus, there is a need to develop novel inhibitors targeting FLT3.

In summary, we identified a novel FLT3 inhibitor, **7c**, as an anti-AML agent that inhibited FLT3 signaling in both in vitro kinase and cell-based assays. Treatment with **7c** downregulated HR and NHEJ repair genes, leading to the inhibition of growth and induction of apoptosis in FLT3-ITD^+^ AML cells. Besides, the combination of **7c** and olaparib significantly inhibited cell proliferation. Overall, our findings suggest that **7c** could be a potential anti-leukemic agent.

## 4. Materials and Methods

### 4.1. Chemistry

All chemical reagents were commercially available and used without further purification. Melting points were determined using the M5000 Melting Point apparatus (Kruss, Hamburg, Germany) and used without any correction. Proton nuclear magnetic resonance (NMR) spectra were recorded using the Avance-500 spectrometer (Bruker, Billerica, MA, USA) at 500 MHz. Chemical shifts are reported in ppm, and Me4Si was used as the reference standard. Mass spectra were recorded using JMS-600W VG Trio-2 GC-MS (Jeol, Akishima, Japan). Reaction products were purified by flash column chromatography using silica gel 60 (230–400 mesh) and monitored by thin-layer chromatography with precoated silica gel 60 F254 (Merck, Darmstadt, Germany). Spots were visualized under UV light (254 nm) after staining with PMA or Hanessian solution.

*5-(4-Fluorophenyl)-N-phenyloxazol-2-amine* (**7c**). Pale yellow solid, mp = 160–162 °C, ^1^H NMR (300 MHz, DMSO-d_6_) δ 10.31 (s, 1H), 7.57–7.68 (m, 5H), 7.26–7.35 (m, 4H), 6.89–7.03 (m, 1H); MS (FAB) *m*/*z* 254 (M^+^).

### 4.2. General Synthesis Procedure

Azide was produced as previously described [21]. Briefly, phenacyl bromide (1.0 eq) was added dropwise to a solution of sodium azide (3.0 eq) in a 3:1 mixture of acetone and water. The resulting mixture was stirred at room temperature for 16 h. The reaction mixture was diluted with water and extracted with ethyl acetate. The combined organic layer was washed with brine, dried over sodium sulfate, and concentrated in vacuo to generate the product (93% yield). Isothiocyanate was produced as previously described [22]. Briefly, thiophosgene (1.2 eq) was slowly added to a cold solution (0 °C) of amine (1.0 eq) in dichloromethane (0.3 M) and stirred at 0 °C for 20 min. The resulting mixture was stirred at room temperature for 4 h, diluted with a saturated solution of K_2_CO_3_, and extracted with DCM several times. The combined organic layers were dried over anhydrous sodium sulfate, filtered, and concentrated in vacuo to generate the product (86% yield). Isooxazole was produced as previously described [23]. Briefly, PPh_3_ (1.0 eq) was added to a solution of azide (1.0 eq) and isothiocyanate (1.0 eq) in dioxane (0.2 M). The resulting mixture was heated to 90 °C and stirred for 4 h. The solvent was cooled at room temperature and evaporated under reduced pressure. The residue was purified by flash column chromatography over silica gel with EtOAc/hexane (1:10) to generate the product (32% yield).

### 4.3. Cell Culture

Human AML cell lines, Molm-13, MV4-11, and HL-60 were maintained in Roswell Park Memorial Institute (RPMI)- 1640 (Molm-13; Welgene, Gyeongsan, Korea) and Iscove’s Modified Dulbecco’s Medium (IMDM) (MV4-11, HL-60; Gibco, Waltham, MA, USA) media supplemented with 10% fetal bovine serum (Welgene) and 1% penicillin–streptomycin (Gibco). Culture flasks were incubated at 37 °C, 5% CO_2_, and 95% relative humidity.

### 4.4. Tumor Xenograft Mouse Model

MV4-11 cells were resuspended in 10% Matrigel for tumor solidification and subcutaneously (s.c.) implanted into the right hind leg thigh of 6-week-old BALB/c nu/nu mice. When the tumor volume reached approximately 80 mm^3^, compound **7** (**7c**; 10 mg kg^−1^) was administered intraperitoneally (i.p.) once every 2 day for three times. Tumor size was measured twice a week using vernier calipers and tumor volume calculated as follows:

Tumor volume = (longest tumor axis × shortest tumor axis)^2^/2 mm^3^.

All animal experiments were conducted in accordance with institutional guidelines and were approved by the Institutional Animal Care Use Committee (IACUC; KIRAMS-BT15-029).

### 4.5. Cell ViabIlity Assay

Cell viability was determined using the Cell Counting Kit-8 (CCK-8) (Dojindo, Japan). Molm-13, MV4-11, and HL-60 were seeded in 96-well plates at a density of 8 × 10^2^, 2 × 10^3^, and 1.2 × 10^3^ cells per well, respectively. Experimental groups were treated with **7c** and incubated at 37 °C and 95% relative humidity for 5 d, whereas the control groups were treated with dimethyl sulfoxide (DMSO) and incubated under the same conditions. CCK-8 solution was added to each well with cells and the absorbance of the sample was measured at 450 nm using a 96-well plate reader (Multiskan EX; Thermo Lab Systems, Waltham, MA, USA).

### 4.6. Western Blotting

Proteins were extracted from cell lysates using TNN (40 mM Tris-HCl (pH 8.0), 0.2% NP-40, 120 mM NaCl) buffer or RIPA lysis buffer (Millipore, Burlington, MA, USA) supplemented with protease inhibitor (Thermo Fisher Scientific, Waltham, MA, USA). Proteins samples, loaded at equal amounts, were separated by sodium dodecyl sulfate polyacrylamide gel electrophoresis on 6–15% gels, and then transferred on to nitrocellulose membranes (Bio-Rad, Hercules, CA USA). The membranes were washed and blocked with 5% skim milk in TBS-T (150 mM NaCl, 10 mM Tris, 0.2% Tween 20), and then incubated overnight with primary antibodies at 4 °C. Secondary antibodies conjugated to peroxidase and visualized immunoreactive proteins enhanced by ECL reagent were detected using ImageQuan LAS 4000 mini (GE Healthcare, Amersham, UK).

### 4.7. Quantitative Real-Time Polymerase Chain Reaction (qRT-PCR)

The total RNA was extracted from the cell lysate using TRIzol^®^ (Thermo Fisher Scientific, USA) to synthesize cDNA using CycleScript reverse transcriptase (Bioneer, Daejeon, Korea). cDNA was mixed with target sequence primers and Maxima SYBR Green/Fluorescein qPCR Master Mix (Thermo Scientific, USA). The sequence of the designed primers targeting DNA repair genes is as follows: *BRCA1*, forward 5′-CTGAAGACTGCTCAGGGCTATC-3′, reverse 5′-AGGGTAGCTGTTAGAAGGCTGG-3′; *BRCA2*, forward 5′-GGCTTCAAAAAGCACTCCAGATG-3′, reverse 5′-GGATTCTGTATCTCTTGACGTTCC-3′; *RAD51*, forward 5′-CTCAGCCTCCCGAGTAGTTG-3′, reverse 5′-CATCACTGCCAGAGAGACCA-3′; *BARD1*, forward 5′-GCCAAAGCTGTTTGATGGAT-3′, reverse 5′-CGAACCCTCTCTGGGTGATA-3′; *XRCC4*, forward 5′- CATTGTTGTCAGGAGCAGGA-3′, reverse 5′-TCTGCAGGTGCTCATTTTTG-3′; *XRCC5*, forward 5′-CGACAGGTGTTTGCTGAGAA-3′, reverse 5′-TCACATCCATGCTCACGATT-3′; *XRCC6*, forward 5′-AAAAGACTGGGCTCCTTGGT-3′, reverse 5′-TGTGGGTCTTCAGCTCCTCT-3′. 18S ribosomal RNA was used as a housekeeping gene to normalize samples. The housekeeping gene sequence is as follows: forward 5′-CACGCCAGTACAAGATCCCA-3′, reverse 5′-TTCACGGAGCTTGTCCA-3′. The conditions of qRT-PCR were as follows: initial duration at 95 °C for 10 min, followed by 50 cycles at 95 °C for 15 s, 60 °C for 30 s, and 72 °C for 30 s. The PCR products were detected and analyzed using the LightCycler 96 system (Roche, Basel, Switzerland). The mRNA level was indicated as the fold change relative to the control. Experiments were carried out in triplicate.

### 4.8. In Vitro Kinase Assay

In vitro kinase profiling of the 11 synthesized compounds was performed by Reaction Biology Corporation (Malvern Panalytical, Malvern, UK).

### 4.9. Molecular Docking Study

Ligand preparation and optimization were conducted using the Prepare Ligands module of Discovery Studio 2019 (Biovia, San Diego, CA, USA). The FLT3 protein structure, as described previously [15], was downloaded from the RCSB-PDB database (http://www.rcsb.org). Before docking, the original crystal ligand quizartinib (AC220) and water molecules were removed from the protein–ligand complexes. Hydrogen atoms were added using the CHARMm force field and the Momany–Rone partial charge as default settings in Discovery Studio 2019. The ligand-binding site was extracted from RCSB-PDB and designated as active site 1. Docking analysis of compounds with the FLT3 protein in the presence of quizartinib was performed using the CDOCKER module. The number of generated poses was set at 100 for each ligand, whereas default settings were selected for other parameters.

### 4.10. Combination Index (CI)

CI scores were calculated using CompuSyn (ComboSyn, Paramus, NJ, USA) after single- and paired-compound treatment. The CI of the two compounds was calculated as follows:

$$\mathrm{CI}\;=\frac{\left(D\right)A}{\left(\mathrm{Dx}\right)A}+\frac{\left(D\right)B}{\left(\mathrm{Dx}\right)B}$$

### 4.11. Statistical Analysis

Data are presented as mean ± standard deviation or standard error. Analysis of variance in conjunction with Student’s *t*-test was carried out to identify significant differences at *p <* 0.05.

## Figures and Tables

**Table 1:** Inhibitory activities of 11 synthesized compounds (100 nM concentration) on FMS-like tyrosine 3 (FLT3) kinases and cell growth.

Compound Identity	Structure		In Vitro Kinase Inhibition (%)			Cell Growth Inhibition (%)		
	R1	R2	FLT3	FLT3 (D835Y)	FLT3 (ITD)	MV4-11	Molm-13	HL-60
**1c**	Br	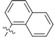	23 ± 4	47 ± 1	63 ± 1	0	0	0
**2c**	Br	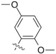	64 ± 9	74 ± 0	82 ± 2	24 ± 5	34 ± 3	0
**3c**	Br	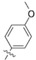	0	0	15 ± 2	0	0	0
**4c**	Br	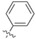	28 ± 2	15 ± 4	57 ± 1	26 ± 2	0	0
**5c**	Br	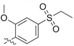	87 ± 0	92 ± 1	89 ± 1	38 ± 4	11 ± 4	0
**6c**	F	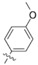	41 ± 6	27 ± 3	66 ± 1	21 ± 5	0	0
**7c**	F	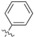	38 ± 6	43 ± 5	70 ± 1	52 ± 1	65 ± 2	0
**8c**	F	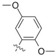	0	0	0	0	0	0
**9c**	F	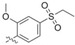	0	0	0	0	0	0
**10c**	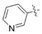	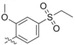	92 ± 0	94 ± 2	92 ± 1	0	0	0
**11c**	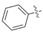	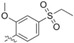	88 ± 1	91 ± 3	85 ± 1	0	0	0

**Table 2:** Half maximal inhibitory concentration (IC_50_) values of compound **7** (**7c**).

Compound Identity	Structure		IC_50_ (nM) (In Vitro Kinase)			IC_50_ of Cell Growth (nM)		
	R1	R2	FLT3	FLT3 (D835Y)	FLT3 (ITD)	MV4-11	Molm-13	HL-60
7c	F	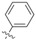	207.2 ± 3.4	215.3 ± 4.1	32.5 ± 2.5	95.51 ± 1.16	61.9 ± 2.45	>10,000

## Supplementary Materials

The following are available, Figure S1: Compounds **2c**, **3c**, **4c**, **5c**, **6c**, **10c**, and **11c** slightly inhibited growth of AML cell lines; Table S1: In silico physicochemical properties of four synthesized compounds compared with quizartinib.
