# Supplementary material for: Discovery of Oxazol-2-amine Derivatives as Potent Novel FLT3 Inhibitors
Source: Molecules. 2020 Nov 5;25(21):5154. doi: 10.3390/molecules25215154 (PMC7663913; doi:10.3390/molecules25215154)
Supplement: Supplementary file 1 [file molecules-25-05154-s001.pdf]

Supplementary Materials

Table S1 *In silico* physicochemical properties of four synthesized compounds compared with quizartinib

Compound identity	*In silico* physiochemical parameters	
	**LogD**	**pKa**
5c	4.235	-2.41
7c	3.834	1.97
10c	3.835	-0.3
11c	5.005	-0.3
Quizartinib	5.064	1.74

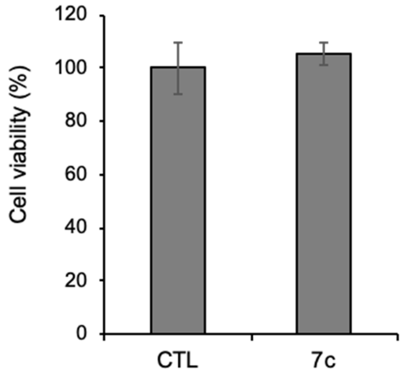

Figure S1. Compound 7c did not inhibit the growth of K562 cells. Human chronic myeloid leukemia K562 cells were treated with 1 μM 7c. After 72 h of treatment, the proliferated cells were analyzed using the CCK-8 assay.

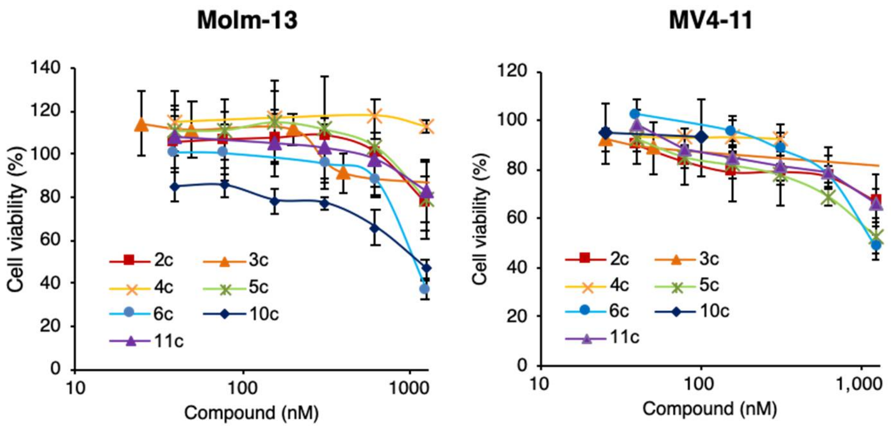

Figure S2. Compounds 2c, 3c, 4c, 5c, 6c, 10c, and 11c slightly inhibited growth of AML cell lines. Human AML cell lines, Molm-13 and MV4-11, was treated with 7 compounds in a concentration range of 40-1000 nM for 5 days, respectively, and viable cells were evaluated by CCK-8 assay.

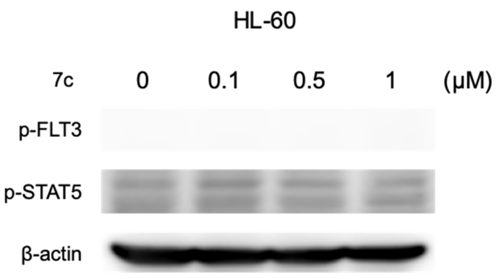

Figure S3. Compound 7c did not induced FLT3 signaling in HL-60. Human FLT3-null AML HL-60 cells were treated with 0.1 - 1 μM 7c for 24 h. The cell lysates were subjected to immunoblotting to detect the indicated proteins. β-actin was used as the loading control.

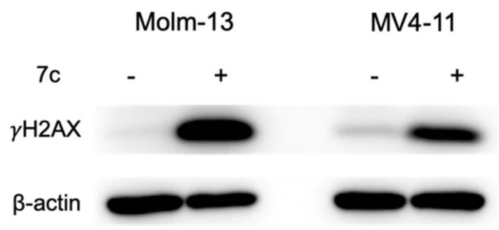

Figure S4. Compound 7c induced DNA damage. Human FLT3-ITD+ AML cells (Molm-13 and MV4-11) were treated with 1 μM 7c for 24 h. The cell lysates were subjected to immunoblotting to detect the indicated proteins. β-actin was used as the loading control.

Supplementary Materials and Methods

General synthetic procedure and chemistry

General synthesis procedure for compounds 1c-11c was the same as for 7c. Chemistry of each compounds were as follows.

5-(4-bromophenyl)-N-(naphthalen-1-yl)oxazol-2-amine (1c)

Yellow solid, mp = 183-185°C, ^1H NMR (300 MHz, DMSO-d_6) δ 10.19 (s, 1H), 8.30-8.34 (m, 1H), 8.10-8.12 (d, *J*=7.32 Hz, 1H), 7.92-7.95 (m, 1H), 7.64-7.67 (m, 3H), 7.48-7.56 (m, 6H); MS (FAB) m/z 365 (M^+).

5-(4-bromophenyl)-N-(2,5-dimethoxyphenyl)oxazol-2-amine (2c)

Yellow solid, mp = 133-135°C, ^1H NMR (300 MHz, DMSO-d_6) δ 10.37 (s, 1H), 7.62-7.65 (m, 4H), 7.52-7.54 (m, 3H), 7.29-7.34 (m, 2H), 6.94-6.98 (t, *J*=8.99 Hz, 1H); MS (FAB) m/z 316 (MH^+).

5-(4-bromophenyl)-N-(4-methoxyphenyl)oxazol-2-amine (3c)

White solid, mp = 213-215°C, ^1H NMR (300 MHz, DMSO-d_6) δ 10.19 (s, 1H), 7.88-7.95 (m, 2H), 7.72-7.76 (d, *J*=2.20 Hz, 2H), 7.64-7.67 (dd, *J*=5.41, 8.71 Hz, 1H), 7.47-7.50 (m, 2H), 6.91-6.94 (m, 2H), 3.74 (s, 3H); MS (FAB) m/z 346 (MH^+).

5-(4-bromophenyl)-N-phenyloxazol-2-amine (4c)

White solid, mp = 214-216°C, ^1H NMR (300 MHz, DMSO-d_6) δ 9.30 (br s, 1H), 7.86 (q, *J*=2.20 Hz, 1H), 7.52-7.85 (m, 5H), 6.93-6.96 (m, 1H), 6.51-6.55 (m, 1H), 3.80 (s, 3H), 3.71 (s, 3H); MS (FAB) m/z 376 (MH^+).

5-(4-bromophenyl)-N-(4-(ethylsulfonyl)-2-methoxyphenyl)oxazol-2-amine (5c)

White solid, mp = 188-190°C, ^1H NMR (300 MHz, DMSO-d_6) δ 10.24 (s, 1H), 8.76-8.77 (dd, *J*=2.20 Hz, 1H), 7.76 (dd, *J*=5.41, 8.71 Hz, 1H), 7.48-7.67 (m, 6H), 7.26-7.28 (m, 1H), 3.97 (s, 3H), 3.21 (q, *J*=7.15 Hz, 2H), 1.11 (t, *J*=7.43 Hz, 3H); MS (FAB) m/z 437 (M^+).

5-(4-fluorophenyl)-N-(4-methoxyphenyl)oxazol-2-amine (6c)

Pale Brown, mp = 198.6-198.8°C, ^1H NMR (300 MHz, DMSO-d_6) δ 10.19 (s, 1H), 8.37 (dd, 2H), 7.53-7.55 (t, *J*=8.99 Hz, 2H) 7.30-7.35 (d, *J*=2.20 Hz, 2H), 7.24-7.27 (m, 2H), 6.91-6.94 (m, 1H), 3.69 (s, 3H); MS (FAB) m/z 346 (MH^+).

N-(2,5-dimethoxyphenyl)-5-(4-fluorophenyl)oxazol-2-amine (8c)

Yellow solid, mp = 214-216°C, ^1H NMR (300 MHz, DMSO-d_6) δ 9.33 (br s, 1H), 7.51-7.72 (m, 6H), 6.91-6.94 (m, 1H), 6.51-6.54 (m, 1H), 3.87 (s, 3H), 3.74 (s, 3H); MS (FAB) m/z 315 (MH^+).

N-(4-(ethylsulfonyl)-2-methoxyphenyl)-5-(4-fluorophenyl)oxazol-2-amine (9c)

Yellow solid, mp = 191-193°C, ^1H NMR (300 MHz, DMSO-d_6) δ 10.14 (s, 1H), 8.78 (d, *J*=2.20 Hz, 1H), 7.65 (dd, *J*=5.41, 8.71 Hz, 2H), 7.46-7.53 (m, 2H), 7.23-7.36 (m, 3H), 3.98 (s, 3H), 3.20 (q, *J*=7.15 Hz, 2H), 1.11 (t, *J*=7.43 Hz, 3H); MS (FAB) m/z 377 (MH^+).

N-(5-(ethylsulfonyl)-2-methoxyphenyl)-5-(4-(pyridin-3-yl)phenyl)oxazol-2-amine (10c)

White solid, mp = 183-185°C, ^1H NMR (300 MHz, DMSO-d_6) δ 10.52 (s, 1H), 9.86 (br s, 1H), 8.68-9.02 (m, 1H), 7.41-7.88 (m, 9H), 7.08-7.38 (m, 1H), 3.83-4.20 (m, 3H), 3.24 (br d, *J*=7.70 Hz, 2H), 1.15 (br t, *J*=7.34 Hz, 3H); MS (FAB) m/z 436 (MH^+).

5-(biphenyl-4-yl)-N-(5-(ethylsulfonyl)-2-methoxyphenyl)oxazol-2-amine (11c)

Yellow solid, mp = 203.0-203.2℃, ^1H NMR (300 MHz, DMSO-d_6) δ 10.52 (s, 1H), 9.81 (s, 1H), 8.70-8.84 (m, 1H), 7.18-7.85 (m, 11H), 3.90-4.06 (m, 3H), 3.14-3.28 (m, 2H), 1.05-1.18 (m, 3H); MS (FAB) m/z 435 (MH^+).
